# Retrospective Perceptions of Income Inequality, School, and Neighborhood Conditions: Associations with Peer Victimization During Adolescence and Young Adulthood

**DOI:** 10.3390/bs16020237

**Published:** 2026-02-07

**Authors:** Joseph Cino, Sierra Barnes, Ann H. Farrell, Mollie J. Eriksson, Tracy Vaillancourt

**Affiliations:** 1 Department of Psychology, Brock University, St. Catharines, ON L2S 3A1, Canada; jc22yd@brocku.ca; 2 Department of Child and Youth Studies, Brock University, 1812 Sir Isaac Brock Way, St. Catharines, ON L2S 3A1, Canada; sb21fs@brocku.ca; 3 Department of Psychology, Neuroscience & Behaviour, McMaster University, 1280 Main Street West, Hamilton, ON L8S 3K1, Canada; erikssom@mcmaster.ca; 4 Counselling Psychology, Faculty of Education, University of Ottawa, 145 Jean-Jacques-Lussier, Ottawa, ON K1N 6N5, Canada; tracy.vaillancourt@uottawa.ca; 5 School of Psychology, Faculty of Social Sciences, University of Ottawa, 145 Jean-Jacques-Lussier, Ottawa, ON K1N 6N5, Canada

**Keywords:** bullying victimization, indirect aggression, adolescence, young adulthood, income inequality, school climate, neighborhood violence

## Abstract

Several immediate and distal social environmental factors work directly and indirectly with one another to contribute to multiple forms of peer victimization. Bullying is the most prevalent form of peer victimization during adolescence; however, peer victimization typically takes the form of indirect aggression during young adulthood. Therefore, we examined how perceptions of school and neighborhood income inequality worked through perceptions of school climate, neighborhood violence, and neighborhood distrust to predict retrospective adolescent bullying victimization and current young adulthood indirect peer victimization. In a cross-sectional sample of 460 young adults (*M_age_* = 20.2, *SD_age_* = 2.18; 59.6% women; 40.4% men; 51.6% White), path analyses revealed that higher school income inequality indirectly predicted higher levels of bullying and indirect peer victimization through lower school climate. Higher neighborhood income inequality also indirectly predicted higher levels indirect peer victimization through higher neighborhood violence. Our findings highlight the importance of targeting adverse environmental risk factors to prevent and intervene in multiple forms of peer victimization across development.

## 1. Introduction

Experiences of peer victimization involve being the target of some form of aggressive behavior (e.g., physical violence, social exclusion, etc.; Graham, 2006; Troop-Gordon, 2017). Given that individuals are increasingly using online platforms to form and sustain peer relationships, it is important to note that peer victimization can occur in both offline and online contexts (Vaillancourt, 2026). Some researchers have considered peer victimization via online platforms (i.e., cybervictimization) distinct from peer victimization that occurs offline (Kasturiratna et al., 2025). However, there is substantial overlap between experiences of online and offline peer victimization (Modecki et al., 2014; Thériault-Couture et al., 2026). Thus, we consider online spaces as one of many contexts in which peer victimization can occur. Although peer victimization is prominent throughout the lifespan, the specific forms of peer victimization that individuals are most likely to experience differ across developmental stages (Troop-Gordon, 2017). For example, bullying is a form of peer victimization that peaks during adolescence and can include physical, verbal, social, and cyber behavior (Barker et al., 2008; Farrell & Vaillancourt, 2021). Bullying also occurs in the context of a power imbalance, whereby the perpetrator yields significant power over the target (Olweus, 1994). Researchers have suggested that bullying peaks during this developmental period because adolescents are increasingly concerned with popularity, and bullying can be an effective means for achieving this elevated social status (Pouwels et al., 2018; Vaillancourt et al., 2003; Wiertsema et al., 2023).

Another form of peer victimization is indirect in nature. It occurs when individuals manipulate peer relationships to harm others through actions such as social exclusion and spreading malicious rumors (Crick, 1997; Vaillancourt, 2013). Unlike bullying, which tends to decrease in prevalence after adolescence (Vaillancourt et al., 2023), individuals continue to experience indirect victimization well into young adulthood (Vaillancourt & Farrell, 2021). Researchers have suggested that adults primarily use indirect aggression relative to other forms of aggression as it is tolerated by society and there are fewer consequences for engaging in this behavior, unlike physical aggression which is strongly discouraged and reprimanded (Björkqvist, 1994; Lagerspetz & Björkqvist, 1994). Consequently, while bullying across forms (e.g., indirect, physical, verbal) is common during adolescence, peer victimization often takes the form of indirect aggression during adulthood (Vaillancourt & Brittain, 2024; Vaillancourt & Farrell, 2021). Although indirect aggression is often expressed within bullying dynamics rather than distinct from them, the consequences for targets are comparably detrimental across forms. For example, extensive research regarding the mental health outcomes for targets of bullying and indirect aggression suggests that these individuals are at increased risk of experiencing anxiety, depression, and conduct problems concurrently (Farrell et al., 2024; Lundh et al., 2014), but also later in life (Lundh et al., 2014; McDougall & Vaillancourt, 2015). Understanding the various risk factors for both bullying victimization and indirect peer victimization is important for reducing these negative mental health outcomes. Research regarding these risk factors suggests that peer victimization may occur under certain social environmental conditions (Hong & Espelage, 2012). Understanding these conditions is therefore essential for preventing peer victimization across developmental stages and, in turn, reducing the associated negative health outcomes. Accordingly, our goal was to examine the social environments in which bullying victimization and indirect peer victimization occur.

### 1.1. Ecological Systems Theory and Peer Victimization

Bronfenbrenner’s ecological systems theory provides a useful framework for understanding the risk factors for bullying victimization and indirect peer victimization. Bronfenbrenner (1979) suggests that human development is not only influenced by individual-level factors (e.g., personality), but also by several nested ecological systems ranging from immediate environments (i.e., micro-system), to broader social systems and cultural norms (i.e., macro-system). Moreover, these ecological systems do not operate in isolation; rather, they influence one another directly and indirectly to shape human development (Bronfenbrenner, 1979). Therefore, when studying risk factors for peer victimization, it is helpful to examine a variety of distal and immediate environmental factors that can contribute to youth experiences, such as the broader economic conditions and immediate school and neighborhood environments.

Most research examining ecological risk factors involved in peer victimization focuses exclusively on bullying victimization, and very few studies have examined the role of ecological factors of indirect peer victimization. Moreover, these studies vary in how they operationalize ecological factors. Researchers examining the role of more immediate environments in bullying victimization (e.g., family, school, neighborhood) have often relied on self-report measures. For example, L. Wang et al. (2023) showed that early adolescents who perceived higher levels of family financial stress were at greater risk of being bullied. Multiple studies have utilized self-report measures to demonstrate that students who perceived higher levels of school connectedness, greater support from teachers, and an overall positive school climate were less likely to be victimized by peers (Acosta et al., 2019; Eugene et al., 2021; W. Wang et al., 2014). Researchers attempting to understand neighborhood factors involved in peer victimization have also heavily relied on self-report measures. To illustrate, M. S. Wang et al. (2019) found that poorer perceptions of neighborhood quality and greater perceptions of neighborhood crime were associated with increased bullying victimization among middle school students in Taiwan. Furthermore, Lee et al. (2021) showed that adolescents who perceived higher levels of disorganization within their neighborhoods were more likely to be bullied. Thus, it is evident that substantial research has focused on how perceptions of immediate environments are related to peer victimization.

However, the same cannot be said for studies looking at how distal environmental factors are related to peer victimization. Specifically, researchers studying the distal environmental factors involved in bullying victimization have primarily relied on regional measures. For example, research examining the relation between income inequality and bullying victimization often measures income inequality using the “Gini index,” which quantifies the amount of income inequality in each region or country based on net household income (Elgar et al., 2009, 2019; Pabayo et al., 2022). Although these studies are informative, they do not fully capture the environmental contexts in which peer victimization occurs. Specifically, Bronfenbrenner (1979) argued that a critical aspect of any ecological system is how individuals *perceive it*, as perceptions of social environments shape human behavior. Thus, it is important to consider how people perceive their social environments to fully understand the ecological risk factors involved in peer victimization. In the present study, we examined whether perceptions of several distal (i.e., school and neighborhood income inequality) and immediate (i.e., school climate, neighborhood violence and neighborhood social trust) social environmental variables were associated with bullying victimization and indirect peer victimization.

### 1.2. Income Inequality and Peer Victimization

Research on the relation between income inequality and peer victimization has primarily focused on bullying victimization (Runions et al., 2025), with no studies to our knowledge looking at income inequality and indirect peer victimization. Research on bullying victimization has consistently shown positive associations with income inequality. These studies typically examine income inequality at the country-level, where countries with higher income inequality (measured by the “Gini index”) have higher rates of bullying victimization (Due et al., 2009; Elgar et al., 2013, 2019). Only a few studies have looked at school or neighborhood income inequality with bullying victimization. For example, a recent study from Japan examined self-reports of socioeconomic status (SES) and bullying victimization among adolescent students and found that the further students were from the average SES within their school, the more likely they were to report being bullied (Sanada, 2025). These findings suggest that students may be more likely to experience peer victimization at schools with higher levels of income inequality. Pabayo et al. (2022) examined the relation between regional income inequality within four Canadian provinces and bullying victimization and found that adolescents living in regions with higher levels of income inequality were more likely to experience bullying victimization. Theoretical explanations for these findings typically involve two separate but related mechanisms. First, high levels of income inequality can foster competitive status dynamics by intensifying dominance hierarchies, in which a small number of individuals hold a disproportionate share of social power (Wilkinson & Pickett, 2017). Second, high levels of income inequality can erode social cohesion because inequality creates a sense of social stratification, which can lead individuals to become disconnected and less trusting of one another (Kawachi et al., 1999). It is also likely that competitive status dynamics created by high levels of income inequality reduce social cohesion and trust (Wilkinson & Pickett, 2017). Consequently, it is not surprising that individuals are more likely to use violence, manipulate, and bully others to compete for social status in contexts where income inequality is high.

The studies outlined above indicate that school and neighborhood income inequality are related to peer victimization, but they are limited for two reasons. First, they only examine the relation between income inequality and adolescent bullying victimization and do not focus on other forms of peer victimization, despite indirect peer victimization being prevalent during young adulthood (Vaillancourt & Brittain, 2024). Second, these studies do not explore how individual perceptions of income inequality are associated with bullying victimization. This is an important gap as people often perceive different levels of income inequality than what exists when measuring inequality by household income (Hauser & Norton, 2017). For example, individuals may perceive high levels of income inequality despite living in a neighborhood or attending a school where not much exists. Therefore, regardless of what is “objectively” present in an environment, perceptions of this environment uniquely shape experience. Furthermore, as Bronfenbrenner (1979) theorized, perceptions of income inequality exert a significant influence on individual-level attitudes and behavior (Hauser & Norton, 2017). To illustrate, in one study, participants were asked to choose from one of five diagrams that they thought best represented the distribution of income inequality in their country (i.e., perceived income inequality), as well as a question regarding their support for wealth redistribution policies (Gimpelson & Treisman, 2018). The researchers found that perceived income inequality predicted support for wealth redistribution policies; however, objective income inequality (i.e., country Gini index) did not.

One mechanism that may link perceived income inequality to peer victimization is heightened status anxiety. Wilkinson and Pickett (2017) theorize that income inequality can lead to high levels of status competition, which in turn can contribute to individuals becoming increasingly concerned about their own social status. More recently, researchers have argued that this claim should be qualified to include perceived income inequality rather than strictly objective measures of inequality (García-Sánchez et al., 2024; Willis et al., 2022). They explain that researchers have consistently demonstrated that higher levels of perceived income inequality predict higher levels of status competition and status anxiety; however, research examining this relation using objective measures of income inequality (e.g., Gini index) have yielded mixed results. Consequently, it may be that perceptions rather than objective levels of income inequality are what drive individuals to become concerned about their social standing (García-Sánchez et al., 2024; Willis et al., 2022). Importantly, status anxiety may be a risk factor for peer victimization. For example, Pozzoli and Gini (2021) showed that adolescents with lower self-perceived social status (i.e., felt they were disliked) were more likely to be victimized by peers. Thus, it is likely that perceived income inequality is also positively related to peer victimization. That said, further investigation is warranted as this hypothesis has yet to be empirically tested.

Finally, indirect effects in the relation between income inequality and peer victimization have been explored in only a few studies (Runions et al., 2025). For example, Elgar et al. (2013) found that national homicide rates partially mediated the relation between country-level income inequality and bullying victimization. Similarly, Pabayo et al. (2022) found that school connectedness and psychological well-being partially mediated the relation between district-level income inequality and bullying victimization. However, no studies to our knowledge have examined how perceptions of income inequality might work indirectly through school and neighborhood factors to predict multiple forms of peer victimization. Consequently, we explored this possibility in the present study.

### 1.3. The Indirect Role of School/Neighborhood Factors

Given that income inequality can promote status hierarchies, erode social cohesion, and increase competition (Kawachi et al., 1999; Wilkinson & Pickett, 2017), it is likely that perceptions of broader income inequality can negatively impact the perceptions of various aspects of more immediate social environments. This is consistent with ecological systems theory, as Bronfenbrenner suggested that social environments from nested systems influence one another indirectly to shape development (Bronfenbrenner, 1979). Two prominent ecological factors that may be negatively affected by income inequality are the school and neighborhood.

For school factors, evidence suggests that income inequality is associated with a poorer school climate. This has been demonstrated in multiple studies where researchers have shown that regional income inequality is related to higher levels of school violence, a decreased sense of school belonging, and lower levels of cooperation among students (Contreras et al., 2015; King et al., 2022; Sommet et al., 2023). Furthermore, researchers have consistently shown that students attending schools with a poorer school climate are at heightened risk of experiencing several forms of peer victimization (physical, verbal, indirect, cyber: O’Brennan & Furlong, 2010; W. Wang et al., 2014). Overall, this suggests that school-level income inequality can negatively impact school climate, which in turn can contribute to higher bullying victimization and indirect peer victimization. However, this hypothesis has yet to be tested.

Regarding neighborhood factors, research has shown that income inequality impacts two different aspects of a neighborhood. The first is neighborhood social trust, which, not surprisingly, tends to be significantly lower in neighborhoods with higher levels of regional income inequality (Kanitsar, 2022; Graafland & Lous, 2019). Moreover, several studies have shown that lower levels of neighborhood trust are a risk factor for multiple forms of peer victimization. For example, Choi et al. (2021) found that lower levels of neighborhood trust were associated with higher levels of bullying victimization. Similarly, Maimon and Browning (2012) showed that lower levels of neighborhood trust predicted increased violence victimization.

The second neighborhood factor associated with income inequality is neighborhood violence. Researchers have suggested that because income inequality creates cultures of competition, individuals are more likely to resort to violence to compete for limited resources in societies where inequality is high (Elgar et al., 2009; Kawachi et al., 1999). Furthermore, multiple studies have shown that neighborhood violence is positively associated with various forms of peer victimization. For example, in one study, youth in South Korea who perceived their neighborhood as unsafe were more likely to report both direct and indirect cyberbullying victimization (Hong et al., 2018). These findings suggest that neighborhood income inequality can work through reduced neighborhood trust and higher levels of neighborhood violence to predict bullying victimization and indirect peer victimization. However, more research is needed to directly test this hypothesis.

### 1.4. Current Study

To our knowledge, the relation between multiple contexts of *perceived* income inequality and peer victimization have not been examined but can be helpful for understanding and preventing multiple forms of victimization across development. Consequently, our first goal was to examine these associations. In a sample of undergraduate students from a university in southern Ontario, Canada, we measured perceptions of retrospective school and neighborhood income inequality during adolescence. We also measured retrospective bullying victimization during adolescence and current indirect peer victimization during young adulthood. Our goal in measuring these forms of peer victimization was to examine whether the same environmental risk factors for bullying victimization during adolescence would be observed for indirect peer victimization during young adulthood. We hypothesized that: (1) Students who perceived higher retrospective levels of school income inequality during adolescence would report experiencing higher retrospective adolescent bullying victimization and explored whether this would extend to current young adult indirect peer victimization. (2) We expected that students who perceived higher levels of retrospective neighborhood income inequality during adolescence would report experiencing higher retrospective bullying victimization and explored whether this would extend to current young adult indirect peer victimization.

Next, given the lack of research examining indirect effects between income inequality and peer victimization (Runions et al., 2025), our second goal was to explore the indirect roles of retrospective neighborhood trust, neighborhood violence, and school climate during adolescence. We hypothesized that: (3) Students who perceived higher levels of neighborhood income inequality would perceive higher levels of social distrust within their neighborhood and consequently be more likely to report bullying victimization and indirect peer victimization. (4) We expected that students who perceived higher levels of neighborhood income inequality would also perceive higher levels of neighborhood violence and consequently be at greater risk of experiencing bullying victimization and indirect peer victimization. (5) We hypothesized that students who perceived higher levels of school income inequality would also perceive lower school climate and consequently be more likely to report bullying victimization and indirect peer victimization. Finally, gender was examined as a moderator, given evidence of differences in bullying victimization and indirect peer victimization between men/boys and women/girls. Specifically, prevalence rates of bullying victimization are higher for boys, whereas prevalence rates of indirect peer victimization are higher for girls (Card et al., 2008; de Bruyn et al., 2010; Lundh et al., 2014; Turner et al., 2013). Thus, we expected that: (6) Our hypothesized effects for bullying victimization would be stronger for men than women, while our hypothesized effects for indirect peer victimization would be stronger for women than men.

Despite relying on several retrospective measures, we aimed to make three novel contributions to the existing literature on peer victimization. First, we aimed to extend previous research on objective income inequality by examining how *perceptions* of income inequality at the school and neighborhood level are related to peer victimization. Second, we used two victimization outcomes (i.e., adolescent bullying victimization and young adulthood indirect peer victimization) to understand whether they shared the same environmental risk factors. Lastly, we simultaneously tested the indirect role of school climate, neighborhood trust, and neighborhood violence to discern their unique contributions to peer victimization at multiple developmental stages.

## 2. Materials and Methods

### 2.1. Participants

Participants were from the Social Relationships and Mental Health study which occurred in April 2023 at a university in southern Ontario. The initial sample was 507, but after excluding 32 individuals (6.3%) due to invalid responses, the final sample resulted in 475 participants (*M_age_* = 20.2 years; *SD* = 2.18; women: *n* = 274, 57.7%; men: *n* = 186, 39.2%; gender diverse: *n* = 13, 2.7%; missing: *n* = 2, 0.4%). Most participants identified as White (*n* = 243; 51.6%) followed by South Asian (*n* = 77; 16.2%), Black (*n* = 51; 10.7%), Mixed (*n* = 37; 7.8%), East Asian (*n* = 22; 4.6%), Southeast Asian (*n* = 16; 3.4%); Latinx (*n* = 9; 1.9%), Middle Eastern or West Asian (*n* = 8; 1.7%), Indigenous (*n* = 2; 0.4%), and Other (*n* = 4; 0.8%). Remaining participants (*n* = 6, 1.3%) did not provide their race/ethnicity. Due to the small number of gender diverse participants, only women and men were included in the subsequent analyses resulting in a sample size of 460 (women: *n* = 270, 59.6%; men: *n* = 186, 40.4%).

### 2.2. Procedure

Following ethics clearance by the relevant university ethics boards, the research team set up a table in the university campus hallway to advertise the study. Participants who were interested scanned a QR code on their personal devices, which linked to the informed consent and study questionnaires. Participants were told that they could skip any question that they would like and that all responses were confidential. The study took approximately 30 min to complete and participants were debriefed and provided with CAD $5.00 as compensation.

### 2.3. Measures

**Bullying Victimization.** Retrospective bullying victimization during adolescence was measured using a single item adapted from the Vaillancourt and Hymel Bullying Involvement Questionnaire (Vaillancourt et al., 2010a). A definition of bullying was provided, followed by asking: “In high school (Grades 9 to 12), how often were you bullied at school?” Items were rated on a five-point scale (0 = *Not at all* to 4 = *Many times a week*).

**Indirect Peer Victimization.** Current indirect peer victimization during young adulthood was measured using the 35 items from the Indirect Aggression Scale (Forrest et al., 2005). The victimization items specifically asked, “How often have people done the following to you?” (e.g., “Purposefully left me out of activities”). Items were rated on a five-point scale (0 = *Never* to 4 = *Always*). An average score was created and higher scores reflected higher indirect peer victimization (α = 0.98).

**School Climate.** Retrospective perceived school climate during adolescence was assessed using a 14-item adapted version of the Sense of School as a Community Scale (Developmental Studies Centre, 1997). Participants were asked to answer the questions “about social relationships during your high school years BEFORE university” (e.g., “students at my school really cared about each other”). Items were rated on a five-point scale (0 = *Disagree a lot* to 4 = *Agree a lot*). An average score was created and higher scores reflected more positive school climate (α = 0.88).

**Neighborhood Distrust.** Retrospective perceived neighborhood trust during adolescence was assessed using five items adapted from the Social Cohesion and Trust subscale of the Collective Efficacy Scale (Sampson et al., 1997; Sampson & Raudenbush, 1999). Participants were asked to answer the questions “about your neighborhood during your high school years BEFORE university” and how much they agreed with each sentence (e.g., “People in this neighborhood can be trusted”). Items were rated on a five-point scale (1 = *Strongly agree* to 5 = *Strongly disagree*). Items were averaged and higher scores reflected higher neighborhood distrust (α = 0.66).

**Neighborhood Violence.** Retrospective perceived neighborhood violence during adolescence was assessed using a five-item scale adapted from Sampson et al. (1997) and Sampson and Raudenbush (1999). Participants were asked to answer the questions “about your neighborhood during your high school years BEFORE university” (e.g., “a violent argument between neighbors”). Items were rated on a four-point scale (1 = *Never* to 4 = *Often*). Items were averaged and higher scores reflected higher neighborhood violence (α = 0.86).

**Income Inequality.** Retrospective perceived school and neighborhood income inequality during adolescence were assessed using two single items. For school inequality, participants were asked: “In your high school, how much income inequality was there among the students’ families?”. For neighborhood inequality, participants were asked: “In your home neighborhood growing up, how much income inequality was there among families?”. Items were rated on a three-point scale (1 = *A low amount* to 3 = *A high amount*) with higher scores reflecting more income inequality at the school and neighborhood, respectively.

**Control Variables.** Age, race/ethnicity, and household income were used as control variables in the primary analyses. For race/ethnicity, due to the small number of racialized participants, race/ethnicity was recoded into White participants (*n* = 243; 51.2%) and participants with under-represented races/ethnicities (*n* = 226; 47.8%) and missing (*n* = 6; 1.3%). For current household income, participants were asked, “What is your best estimate of the total annual income for your family per year?” Response options included 1 = *Less than $40,000*, 2 = *Between $40,000 and $50,000* and increased in increments of $10,000 until 13 = *More than $150,000*. The median household income was between $90,000 and $100,000.

### 2.4. Analytic Plan

First, descriptive analyses, bivariate correlations, independent samples *t*-tests, and missing value analyses were conducted on SPSS 29. Independent samples *t*-tests were used to examine mean differences in key study variables by gender. Second, the primary analyses were conducted in MPlus 8.1 (Muthén & Muthén, 1998) using full information maximum likelihood estimation (FIML). The primary model involved examining whether perceived school income inequality and perceived neighborhood income inequality predicted bullying victimization and indirect peer victimization indirectly through school climate, neighborhood distrust, and neighborhood violence. Therefore, the model involved estimating direct paths from: (1) school income inequality and neighborhood income inequality to school climate, neighborhood distrust, and neighborhood violence, (2) school climate, neighborhood distrust, and neighborhood violence to bullying victimization and indirect peer victimization, and (3) school income inequality and neighborhood income inequality to bullying victimization and indirect peer victimization. We also allowed school income inequality to covary with neighborhood income inequality, as well as school climate, neighborhood distrust, and neighborhood violence to covary with one another, and finally bullying victimization and indirect peer victimization to covary with each other. Control variables (age, race/ethnicity, household income) were allowed to covary with school income inequality and neighborhood income inequality, and direct paths were estimated from control variables to all other variables. This model was estimated twice to examine gender as a moderator. Using multigroup path modeling, we first estimated a model in which all paths and covariances were freely estimated for men and women. We then compared this model to a second model in which all paths and covariances were constrained to be equal across genders. A significant difference in model fit indicated gender differences.

Model fit was examined using: comparative fit index (CFI) values > 0.95, Tucker–Lewis Index (TLI) values > 0.95, root mean square error of approximation (RMSEA) values < 0.06, and standardized root mean square residual (SRMR) values < 0.08 (Browne & Cudeck, 1992; Hu & Bentler, 1999). We also reported the χ^2^ test of significance, but this test is sensitive to large sample sizes (Kline, 2016). Finally, tests of indirect effects were examined using bootstrapping with 2000 samples and maximum likelihood (ML) estimator to examine 95% confidence intervals. Bootstrapping was used with ML estimator to test for indirect effects as it accounts for the non-normal distribution of the indirect effects. Confidence intervals that did not cross zero were considered to reflect significant indirect effects (Biesanz et al., 2010).

## 3. Results

### 3.1. Descriptive Statistics

The means and standard deviations for the overall sample and for women and men are presented in Table 1. The descriptive statistics revealed no issues with skewness and kurtosis but revealed slight positive skew for bullying victimization. Therefore, maximum likelihood robust estimator (MLR) was used for primary analyses. Independent samples *t*-test revealed no significant mean differences by gender in any of the key study variables. Little’s MCAR test was not significant, χ^2^(213) = 239.047, *p* = 0.106. Therefore, we did not have evidence for rejecting MCAR and proceeded with using FIML. Bivariate correlations are presented in Table 2. Higher bullying victimization and indirect peer victimization were correlated with one another and were both significantly correlated with lower school climate, higher neighborhood violence, and higher neighborhood distrust. Higher indirect peer victimization was also significantly positively correlated with higher neighborhood income inequality. Lower school climate, higher neighborhood distrust, higher neighborhood violence, higher school income inequality, and higher neighborhood income inequality were all significantly correlated with one another with two exceptions. Neighborhood violence and school income inequality were not significantly correlated, and school climate and neighborhood income inequality were not significantly correlated. Among control variables, higher income was significantly correlated with lower neighborhood distrust, school income inequality, and neighborhood income inequality. Being from under-represented racial/ethnic groups was significantly correlated with higher neighborhood distrust, neighborhood violence, and neighborhood income inequality. Finally, being older and from an under-represented race/ethnicity were associated with lower household income.

### 3.2. Primary Analyses

**Figure 1:**
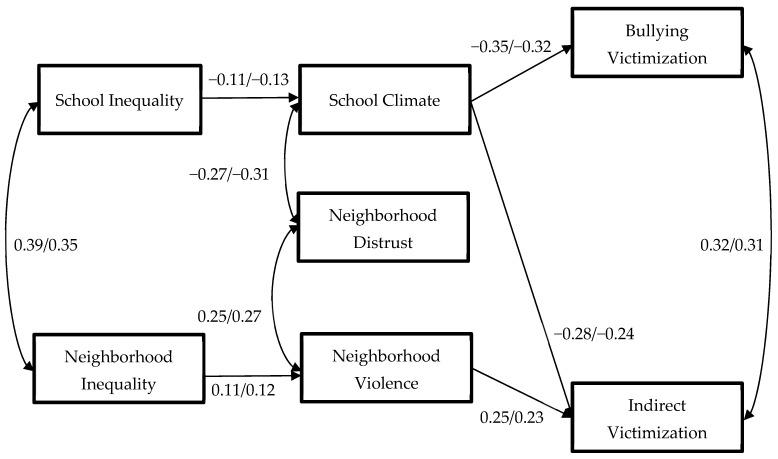
Associations between income inequality, school and neighborhood factors, and victimization. Note: Model is fully constrained between women and men; values represent standardized coefficients or correlations significant at *p* < 0.05; coefficients before slash represent women, coefficients after slash represent men. Control variables and non-significant paths are not included for ease of presentation.

The first path model with all paths and covariances free to vary by gender was saturated and therefore indicators of model fit were not informative. The second path model with all paths and covariances constrained to be equal across women and men had excellent fit and indicated no significant differences by gender, Δχ^2^[Δdf = 45] = 47.349, *p* = 0.377, c. 1.012; RMSEA = 0.015, 90% CI [0.000–0.047]; CFI = 0.990; SRMR = 0.048. Therefore, this model constrained across gender was our final model. See Figure 1 and Table 3 for model results. As expected, higher bullying victimization and indirect peer victimization were both significantly predicted by lower school climate. However, only higher indirect peer victimization was predicted by higher neighborhood violence. Lower school climate was additionally predicted by higher school income inequality, whereas higher neighborhood violence was additionally predicted by higher neighborhood income inequality. For control variables, being younger significantly predicted higher neighborhood violence and being from under-represented racial/ethnic groups over being White predicted higher neighborhood violence. Considering these significant direct paths, we tested the following indirect effects: school income inequality to school climate to (1) bullying victimization and (2) indirect peer victimization, and neighborhood income inequality to neighborhood violence to (3) indirect peer victimization. As expected, higher school income inequality significantly predicted lower school climate, which in turn significantly predicted higher bullying victimization (*b* = 0.06, se = 0.03, β = 0.04, 95% CI [0.009, 0.112]) and indirect peer victimization (*b* = 0.04, se = 0.02, β = 0.03, 95% CI [0.006, 0.079]). Furthermore, higher neighborhood income inequality predicted higher neighborhood violence, which in turn significantly predicted higher indirect peer victimization (*b* = 0.03, se = 0.02, β = 0.03, 95% CI [0.004, 0.071]).

## 4. Discussion

We examined whether perceptions of several distal and immediate social environmental variables were associated with retrospective adolescent bullying victimization and current young adulthood indirect peer victimization. Our first goal was to examine whether perceived income inequality would predict multiple forms of peer victimization. Contrary to hypotheses one and two, perceptions of income inequality at the school and neighborhood level did not directly predict bullying victimization or indirect peer victimization. This finding contradicts several previous studies which showed that regional income inequality (measured by the “Gini index”) was associated with higher levels of bullying victimization (Due et al., 2009; Elgar et al., 2013, 2019; Pabayo et al., 2022). However, our study differs from these studies in two important ways.

First, our study is novel as we examined how *perceptions* of school and neighborhood income inequality were related to multiple forms of peer victimization. This is an important distinction as people often perceive different levels of income inequality than what exists when measuring inequality by household income (Hauser & Norton, 2017). Specifically, when measuring income inequality by household income (i.e., objectively), all households in each region are accounted for equally. By comparison, perceptions of broader income inequality are largely shaped by more immediate social contexts (Hauser & Norton, 2017; Xu & Garand, 2010). For example, students’ perceptions of broader school income inequality may be disproportionately influenced by the level of inequality in the smaller peer group (e.g., classroom) and where they perceive themselves to rank within this peer group. It is possible these contrasting results reflect differences between perceived and regional measures of income inequality. Second, there are few studies that have examined the association between regional income inequality and peer victimization through indirect effects (Elgar et al., 2013; Pabayo et al., 2022). Among these studies, most have focused on testing one indirect pathway at a time as opposed to the multiple that were tested simultaneously in the current study. Not accounting for multiple mediating factors may have contributed to the significant direct effects observed in prior studies. By contrast, we tested for indirect effects through three variables (i.e., neighborhood distrust, neighborhood violence, and school climate) in a single path model, which may help explain the absence of direct effects from income inequality to peer victimization in our study. These three variables accounted for a significant amount of variance in peer victimization as reflected in the results of our second goal of the study.

The second goal of our study was to examine the indirect role of neighborhood distrust, neighborhood violence, and school climate. Contrary to hypothesis three, higher neighborhood income inequality did not predict higher levels of neighborhood distrust, and higher levels of neighborhood distrust did not predict either form of peer victimization. Our fourth hypothesis (i.e., indirect effect of neighborhood violence) was partially supported as higher neighborhood income inequality predicted higher indirect peer victimization through higher levels of neighborhood violence. However, this result did not replicate for bullying victimization as higher levels of neighborhood violence did not predict bullying victimization. It is worth highlighting that neighborhood violence, but not distrust, was related to higher levels of neighborhood income inequality and indirect peer victimization. One reason for this may be that neighborhood violence and neighborhood distrust are not necessarily separate constructs; rather, neighborhood distrust is a consequence of neighborhood violence (Wilkinson & Pickett, 2017). For example, Intravia et al. (2016) showed that greater perceptions neighborhood violence predicted substantially lower levels of neighborhood trust. This result held constant even after controlling for several other factors related to neighborhood trust such as residential stability. The findings from our study also supports this rationale. When examining the bivariate correlations, we observed significant positive associations between neighborhood income inequality and neighborhood distrust and between neighborhood distrust and indirect peer victimization. However, when we allowed neighborhood distrust to covary with neighborhood violence in the final model, these effects were attenuated. This finding suggested that although neighborhood distrust is an important environmental factor involved in peer victimization, exposure to violence in the larger environment might predominantly contribute to individual level victimization. Future research can further examine the mechanisms among neighborhood factors. Our fifth hypothesis that higher school income inequality would indirectly predict increased bullying victimization and indirect peer victimization through a poorer school climate was supported. These findings suggested that perceptions of school income inequality were not directly related to peer victimization, but they might work through perceptions of more immediate environments (i.e., school climate) to predict peer victimization across multiple developmental stages. This pattern was consistent with ecological systems theory, as Bronfenbrenner theorized that social environments influence one another indirectly to shape development (Bronfenbrenner, 1979).

Given that very few studies have explored environmental risk factors for indirect peer victimization, another goal of our study was to examine whether the environmental risk factors for bullying victimization during adolescence replicated for indirect peer victimization during young adulthood. With respect to neighborhood factors, we did not observe the same pattern of direct and indirect effects predicting both forms of peer victimization. Greater perceptions of neighborhood income inequality predicted higher levels of neighborhood violence, which in turn predicted higher indirect peer victimization, but not bullying victimization. However, we did observe the same pattern of direct and indirect effects for school factors predicting both forms of peer victimization. Greater perceptions of school income inequality predicted poorer perceptions of school climate, which in turn predicted higher bullying and indirect peer victimization. These findings indicated that perceptions of the school environment not only shape retrospective experiences of peer victimization during adolescence, but also experiences of current peer victimization during young adulthood. Furthermore, researchers suggest that there are considerable levels of continuity in experiences of peer victimization from adolescence to young adulthood. For example, multiple studies have shown that individuals who are victimized by peers during adolescence are at a heightened risk for experiences of peer victimization in the workplace during young adulthood (Brendgen et al., 2019; Brendgen & Poulin, 2018). Our study provided novel contributions of the developmental continuity from adolescent bullying victimization to indirect peer victimization during young adulthood using a retrospective design, which to our knowledge has not been examined with these social environmental variables. Thus, addressing the risk factors for peer victimization during adolescence, such as broader perceptions of school income inequality and more immediate perceptions of school climate are essential for reducing the prevalence of peer victimization during young adulthood.

It is noteworthy that perceptions of the school environment were related to peer victimization during both adolescence and young adulthood, while perceptions of the neighborhood environment were only related to experiences of peer victimization during young adulthood. This was likely because most of the peer victimization during adolescence takes place at school (Vaillancourt et al., 2010b). Specifically, the school is the environmental context where adolescents spend the most amount of time with peers, making it particularly salient for shaping experiences of peer victimization during this developmental period. This pattern was consistent with prior studies which have shown that the school environment plays a vital role in peer victimization during adolescence. For example, in one meta-analysis, researchers examined various contextual factors related to adolescent bullying victimization including family, school, and community factors and found school climate to have one of the largest effect sizes (Cook et al., 2010). By comparison, most young adults are no longer directly involved in their high school environments. Thus, it is likely that young adults’ retrospective perceptions of their high school environments are not the only important ecological factor shaping their current experiences of peer victimization. Instead, their retrospective perceptions of multiple social environments during adolescence (i.e., school and neighborhood) likely contributed to their current experiences of peer victimization. That said, it should be noted that neighborhood factors may shape experiences of peer victimization through indirect pathways that were not explored in this study. For example, neighborhood distrust and neighborhood violence likely influence school climate as schools are typically nested within neighborhoods. Thus, it is possible that perceptions of broader income inequality influence perceptions of neighborhood conditions, which in turn work through more immediate perceptions of school climate to predict peer victimization. Further research is needed to test this hypothesis.

Finally, we expected that our hypothesized effects for bullying victimization would be stronger for men than women and our hypothesized effects for indirect peer victimization would be stronger for women than men. However, we did not observe any differences in the strength of these effects based on gender. With respect to bullying victimization, this may be because the measure of bullying victimization used in the current study was fairly generalized, grouping multiple forms of bullying into a single item (e.g., physical, verbal, indirect, cyber). Gender differences may be more apparent if different forms of bullying were separated as gender differences in bullying victimization are primarily found across subtypes (Carbone-Lopez et al., 2010; Salmon et al., 2018; Scheithauer et al., 2006). In terms of indirect peer victimization, although research suggests that indirect aggression is higher in girls than boys, the effect size of this difference is small (Archer, 2004; Card et al., 2008).

### 4.1. Limitations and Future Directions

There are several limitations to our study. First, we used a sample of undergraduate students. Consequently, our sample was likely restricted with respect to measures of inequality and violence, as students attending post-secondary institutions are from predominantly affluent backgrounds (Frempong et al., 2012). Despite our restricted sample, we were able to find significant effects from school and neighborhood income inequality, school climate, and neighborhood violence to multiple forms of peer victimization. This indicates that our results should generalize to more samples that are diverse with respect to inequality and violence potentially by recruiting samples from a range of geographic regions, with the possibility of stronger effects. However, further research is needed to replicate our findings in the larger population.

The second set of limitations relates to the cross-sectional nature of our study. Although our model is grounded in theory and previous empirical work, we recognize that a cross-sectional study cannot address causal conclusions. There are likely several statistically equivalent models to the one we have tested (MacCallum et al., 1993) and true experimental designs are needed to draw causal conclusions. However, future longitudinal studies can address temporal pathways. Given that longitudinal studies require substantial resources, our study is an important first step for establishing these patterns cross-sectionally. A related concern is that we used several retrospective measures due to the cross-sectional nature of our study, which suggests that our results may be subject to recall bias. Other variables that we did not account for such as current psychological state, negative affect, level of parental support, and/or personality profile may have impacted how participants perceived their social environments and recalled experiences of peer victimization. For example, Farrell and Volk (2017) found that adolescents who scored higher on measures of honesty-humility and agreeableness perceived lower levels of school competitiveness and neighborhood violence. Furthermore, Espelage et al. (2016) showed that young adults’ retrospective reports of childhood bullying victimization were positively associated with current levels of depression and anxiety. Therefore, future studies should examine these factors to better understand how they are related to both perceptions of different social environments and recollections of peer victimization. Nevertheless, the use of retrospective measures does not invalidate our results. Extensive research suggests that retrospective measures of environmental conditions (e.g., neighborhood violence) and bullying victimization are valid. For example, Osypuk et al. (2015) examined the validity of retrospective measures of childhood neighborhood conditions in a sample of adult women and found little evidence of recall bias. Furthermore, studies have consistently shown that individuals are able to recall experiences of bullying victimization during childhood and adolescence with a fair level of accuracy (Green et al., 2018; Rivers, 2001). That said, cross-sectional designs are not a replacement for longitudinal research (Rivers, 2001). It is also possible that reflecting on current experiences of indirect peer victimization biased participants’ retrospective reports of bullying victimization. However, this is not likely, as there was only a modest correlation between bullying victimization and indirect peer victimization (*r* = 0.39), suggesting that there was not substantial overlap between these two forms of peer victimization. Moreover, even when controlling for the shared variance between bullying victimization and indirect peer victimization, both forms of peer victimization were independently predicted by some of same social environmental variables (e.g., lower school climate).

Third, our measures of perceived school and neighborhood income inequality present a construct validity limitation as we used single items with only three response options. However, several other researchers have measured perceived income inequality using a single item. For example, Oshio and Urakawa (2014) showed that perceived income inequality was negatively associated with subjective well-being using a single binary variable (i.e., participants either did or did not perceive widening perceptions of income inequality). This result has been replicated several times, indicating that their single item measure was valid (e.g., John et al., 2025; Schmalor & Heine, 2022; Vezzoli et al., 2023). Furthermore, most of the research that has measured perceptions of income inequality has done so at the country level. Few studies have measured perceptions of income inequality at the neighborhood level, and no studies to our knowledge have measured perceptions of income inequality at the school level. Consequently, despite the limitations of our income inequality measure, our study provided important preliminary findings for future research to expand on. Future studies should develop more comprehensive measures of perceived school and neighborhood income inequality to replicate our findings with.

Fourth, our study relied entirely on self-report data indicating that there may be a certain degree of common method variance. However, the use of self-report measures was important for our study as our goal was to understand how individuals’ *perceptions* of income inequality, school climate, neighborhood distrust, and neighborhood violence were related to their own *perceptions* of victimization experiences. Understanding how perceptions of different social environments are related to peer victimization is critical because the way people perceive their social environments uniquely shapes their experiences beyond just the objective qualities of these environments (Bronfenbrenner, 1979). Apart from our single item measures, we also selected measures which have demonstrated good reliability, psychometric properties, and factor structure. Furthermore, the bivariate correlations between our variables were not overly high as no bivariate correlations were greater than *r* = 0.40. That said, our study was only the first step for establishing the relation between perceptions of these social environments and experiences of peer victimization. Future research should test whether our results hold after controlling for additional factors such as regional levels income inequality and neighborhood violence, and peer nominated high school bullying victimization.

Finally, we only examined how perceptions of income inequality were related to peer victimization. Although perceptions of income inequality are an important and understudied factor involved in peer victimization, perceptions alone do not fully capture how income inequality is related to peer victimization. Specifically, measures of perceived income inequality capture individuals’ lived experiences; however, these measures neglect the objective amount of income inequality in each environment. Several previous studies have shown that regional income inequality is also an important factor involved in peer victimization (Due et al., 2009; Elgar et al., 2013, 2019; Pabayo et al., 2022). Thus, future studies should examine how perceived and regional income inequality interact to predict various forms of peer victimization. For example, looking at whether adolescents and young adults who live in regional contexts of low-income inequality but perceive high levels of income inequality are more likely to experience peer victimization.

### 4.2. Implications and Conclusions

Our study has important theoretical implications as it adds to the social ecological understanding of peer victimization. Specifically, previous research has used ecological systems theory as a foundation for examining a variety of environmental factors associated with bullying victimization; however, to our knowledge, researchers have not applied ecological systems theory to examine adult indirect peer victimization. Thus, our study is novel as we extended ecological systems theory to explore the environmental risk factors for indirect peer victimization during young adulthood. Our results revealed that some of the same environmental factors involved in adolescent bullying victimization can also be implicated in indirect peer victimization during young adulthood. These results support the idea of developmental continuity in experiences of peer victimization and highlight the importance of early intervention. Our study also builds on previous research showing that higher levels of regional income inequality (measured by the “Gini index”) are related to higher prevalence rates of bullying victimization (Due et al., 2009; Elgar et al., 2013, 2019; Pabayo et al., 2022). Specifically, our findings showed that the amount of income inequality individuals *perceive* is also an important factor involved in multiple forms of peer victimization. Furthermore, our study adds to existing knowledge by explaining some of the mechanisms that income inequality works through to predict peer victimization. Specifically, we showed that broader perceptions of school income inequality work through more immediate perceptions of school climate to predict bullying victimization and indirect peer victimization. We also demonstrated that broader perceptions of neighborhood income inequality work through more immediate perceptions of neighborhood violence to predict indirect peer victimization.

These findings have important practical implications, especially with respect to prevention efforts. Interventions that aim to reduce the consequences of income inequality at the school level may be particularly effective. For example, improving school cohesion can lead to lower levels of peer victimization by reducing the need for students to compete with one another. This has been demonstrated in multiple studies which have shown that increased school cohesion is related to lower levels of peer victimization (Sapouna, 2010; Springer et al., 2016). The KiVa program is one promising intervention that aims to reduce peer victimization by fostering school-wide norms emphasizing peer defending and the importance bystander intervention, rather than targeting individual students (Salmivalli et al., 2013). In fact, one meta-analysis comparing anti-bullying interventions across the world found the KiVa program to be one of the most effective at reducing peer victimization (Gaffney et al., 2019). Consequently, improving social cohesion among peers may be especially important for preventing peer victimization.

Classroom status hierarchies are another consequence of income inequality that is closely related to peer victimization. For example, Garandeau et al. (2014) showed that higher levels of classroom status hierarchy in the middle of the school year predicted increased bullying victimization at the end of the school year. Teachers play an important role in reducing classroom status hierarchies by creating opportunities for students to foster new friendships (e.g., frequently changing seating charts; Gest & Rodkin, 2011). Thus, efforts to prevent peer victimization should also involve educating teachers about best practices for fostering equal status classrooms. Overall, our findings indicate that the school is a particularly important environmental factor involved in adolescent bullying victimization and indirect peer victimization during young adulthood. Therefore, interventions targeting these school factors (i.e., social cohesion among peers, classroom status hierarchies) not only have the potential to reduce adolescent bullying victimization but can also have important long-term effects by reducing indirect peer victimization into young adulthood.

## Figures and Tables

**Table 1:** Descriptive statistics for study variables overall and by gender.

		Possible Range		Total		Women		Men			Test	
	*n*	Min	Max	*M*	*SD*	*M*	*SD*	*M*	*SD*	*t*	*p*	*d*
Bullying Victimization	456	0	4	0.63	0.91	0.62	0.90	0.64	0.92	−0.23	0.818	−0.02
Indirect Peer Victimization	456	0	4	1.28	0.79	1.29	0.76	1.28	0.84	0.10	0.922	0.01
School Climate	450	0	4	2.19	0.71	2.20	0.76	2.17	0.64	0.56	0.578	0.05
Neighborhood Distrust	451	1	5	2.63	0.63	2.60	0.65	2.67	0.61	−1.23	0.218	−0.12
Neighborhood Violence	456	1	4	1.75	0.75	1.76	0.76	1.74	0.73	0.18	0.861	0.02
School Income Inequality	436	1	3	1.90	0.64	1.93	0.62	1.85	0.66	1.28	0.203	0.13
Neighborhood Income Inequality	436	1	3	1.68	0.68	1.68	0.68	1.68	0.68	0.02	0.985	0.00

Note. Gender coded as 0 = women and 1 = men.

**Table 2:** Correlations of all study variables.

Variables	1	2	3	4	5	6	7	8	9	10
1. Age	-									
2. Income	−0.16 **	-								
3. Race/Ethnicity	0.11 *	−0.28 **	-							
4. Bullying Victimization	0.01	−0.03	0.02	-						
5. Indirect Peer Victimization	0.01	−0.07	0.06	0.39 **	-					
6. School Climate	−0.03	0.04	−0.04	−0.33 **	−0.28 **	-				
7. Neighborhood Distrust	0.06	−0.11 *	0.12 **	0.11 *	0.12 *	−0.31 **	-			
8. Neighborhood Violence	−0.07	−0.09	0.12 **	0.11 *	0.26 **	−0.10 *	0.27 **	-		
9. School Income Inequality	−0.03	−0.13 *	0.01	0.04	0.07	−0.12 *	0.10 *	0.07	-	
10. Neighborhood Income Inequality	0.01	−0.17 **	0.18 **	0.07	0.10 *	−0.05	0.13 **	0.14 **	0.37 **	-

Note. Income = household income; race/ethnicity coded as 0 = White, 1 = Under-represented groups. * *p* < 0.05. ** *p* < 0.01.

**Table 3:** Results of path analysis examining income inequality, school and neighborhood factors, and victimization.

	School Climate			Neighborhood Distrust			Neighborhood Violence			Bullying Victimization			Indirect Peer Victimization		
Independent Variables	*B(se)*	β	*p*	*B(se)*	β	*p*	*B(se)*	β	*p*	*B(se)*	β	*p*	*B(se)*	β	*p*
Age	−0.00(0.01)	−0.01/−0.02	0.787	0.01(0.01)	0.03/0.04	0.462	−0.03(0.02) *	**−0.08/−0.12**	**0.023**	0.01(0.02)	0.01/0.01	0.789	0.01(0.02)	0.02/0.02	0.639
Race ^a^	−0.04(0.07)	−0.03/−0.03	0.575	0.10(0.07)	0.08/0.08	0.115	0.16(0.07) *	**0.10/0.11**	**0.029**	−0.02(0.09)	−0.01/−0.01	0.776	0.00(0.07)	0.00/0.00	0.979
Income	0.00(0.01)	0.02/0.02	0.744	−0.01(0.01)	−0.06/−0.06	0.308	−0.01(0.01)	−0.05/−0.06	0.322	−0.00(0.01)	−0.01/−0.01	0.861	−0.01(0.01)	−0.03/−0.03	0.570
School Inequality	−0.13(0.06) *	**−0.11/−0.13**	**0.026**	0.06(0.05)	0.06/0.07	0.193	0.02(0.06)	0.01/0.02	0.773	−0.02(0.07)	−0.01/−0.01	0.782	0.00(0.06)	0.00/0.00	0.971
Neighbor Inequality	0.02(0.06)	0.02/0.02	0.772	0.07(0.05)	0.08/0.08	0.116	0.12(0.06) *	**0.11/0.12**	**0.031**	0.06(0.07)	0.05/0.05	0.369	0.06(0.06)	0.05/0.05	0.328
School	-			-			-			−0.43(0.07) ***	**−0.35/−0.32**	**<0.001**	−0.30(0.05) ***	**−0.28/−0.24**	**<0.001**
Distrust	-			-			-			−0.03(0.07)	−0.02/−0.02	0.661	−0.06(0.07)	−0.05/−0.05	0.339
Violence	-			-			-			0.09(0.06)	0.08/0.07	0.129	0.26(0.05) ***	**0.25/0.23**	**<0.001**
*R*^2^ Women	0.01(0.01)		0.223	0.03(0.02)		0.050	0.04(0.02) *		**0.027**	0.13(0.03) ***		**<0.001**	0.15(0.04) ***		**<0.001**
*R*^2^ Men	0.02(0.02)		0.221	0.04(0.02) *		**0.039**	0.05(0.02) *		**0.028**	0.11(0.04) **		**0.002**	0.12(0.03) ***		**<0.001**
*N*	460														

Note. ^a^ 0 = White, 1 = Under-represented groups; Income = Household Income; School Inequality = School Income Inequality; Neighbor Inequality = Neighborhood Income Inequality; School = School Climate; Distrust = Neighborhood Distrust; Violence = Neighborhood Violence. Model is constrained to be equal across women and men. For standardized β, coefficients before slash represent women, coefficients after slash represent men. Significant effects bolded for ease of presentation. * *p* < 0.05, ** *p* < 0.01, *** *p* < 0.001.

## Data Availability

The data presented in this study are available upon reasonable request from the corresponding author and are not publicly available due to ethical restrictions.
